# Comply or evade: factors influencing private enterprises’ basic old-age insurance responsibilities

**DOI:** 10.3389/fpubh.2024.1300490

**Published:** 2024-03-04

**Authors:** Wanli Xu, Haosen Ma, Zihao Peng, Caodie Peng, Xihong Qian

**Affiliations:** ^1^School of Politics and Public Administration, South China Normal University, Guangzhou, China; ^2^School of Management, University of Science and Technology of China, Hefei, China; ^3^School of Health Management, Guangzhou Medical University, Guangzhou, China

**Keywords:** basic old-age insurance, private enterprises, enterprise profit, regional development level, labor union, workers’ congress

## Abstract

**Introduction:**

Private enterprises are playing an increasingly important role in production and employment in China. However, due to less regulation and a stronger profit motivation than state-owned enterprises with more standardized management, a considerable portion of these private enterprises fall short of fulfilling their basic responsibilities for government-mandated old-age insurance.

**Methods:**

This study establishes a comprehensive research framework aimed at delving into the precise factors contributing to the lax adherence of private enterprises to their basic old-age insurance obligations. This framework takes into account a range of factors, including enterprise profitability, the external environmental context (specifically the level of regional development), and internal organizational dynamics (such as the presence of labor unions and workers’ congresses). To validate this framework, empirical data from a substantial sample of 3,123 private enterprises, which were part of the 10th Chinese Private Enterprise Survey (CPES), were utilized. This study employs the stepwise multiple regression analysis and conducts robustness tests to ensure the model’s effectiveness.

**Results:**

Enterprise profitability, regional development levels, and the existence of labor unions all wield a positive influence on basic old-age insurance coverage that private enterprises extend to their workforce. Moreover, an intriguing aspect emerges: the developmental stage of the region, as well as the presence of labor unions exercise a negative moderating effect on the relationship between enterprise profitability and the coverage rate of basic old-age insurance. In essence, this implies that the basic old-age insurance coverage rate for private enterprises operating in well-developed regions and those with established labor unions is relatively insulated from fluctuations in profitability.

**Discussion:**

To increase the participation rate of private enterprises’ basic old-age insurance, it is important to improve the overall development environment for private enterprises, enhance internal organizational mechanisms, and strengthen regulatory oversight of enterprises in various regions.

## 1 Introduction

The issue of older adult care transcends individual concerns to become a societal imperative, profoundly impacting a country’s sustained stability and development (1). The establishment and implementation of the fundamental old-age insurance system, henceforth referred to as “old-age insurance,” has played a pivotal role in partially safeguarding the basic living necessities of the older adult while furnishing them with a stable and dependable livelihood source (2, 3). Furthermore, this system fosters the seamless integration of the fresh labor force, facilitates intergenerational labor replacement, and propels the enhancement of social welfare and equitable income distribution (4–6). Nonetheless, the statistics show a stark contrast in the proportion of employees participating in old-age insurance between private enterprises and state-owned or collective enterprises, with the participation rate in the private sector standing at only 48.75 percent. This signifies that the majority of employees in private enterprises are not covered by old-age insurance. Given that private enterprises are an important force driving the development of China’s market economy and employ a large number of laborers (7), neglecting to provide these employees with appropriate old-age insurance may hinder the development of China’s old-age insurance program. Promoting private enterprises to actively fulfill their old-age insurance obligations will not only protect their employees after retirement but also help to maintain social stability.

Studying the factors that affect private enterprises’ fulfillment of their pension obligations is of significant importance. However, existing academic empirical research in this area remains scarce (8) for two main reasons. First, old-age insurance is a fundamental element within the social security framework, and with clear government regulations, enterprises will consciously comply with these regulations. Therefore, there is considered to be no problem for employees to participate in old-age insurance. However, the phenomenon of employees circumventing the purchase of old-age insurance is still serious in many countries because there are still problems in the design and implementation of the system. This phenomenon is particularly common in private enterprises that are less regulated and have stronger profit-seeking incentives than state-owned enterprises with well-regulated management. Second, empirical research in this area requires a large amount of enterprise-level data related to the implementation of employee pension plans. Unfortunately, quantitative studies at this level are still rare, which makes it difficult to determine the exact reasons why private enterprises do not fulfill their old-age insurance obligations (9–11).

This study constructs a comprehensive framework that analyzes two dimensions of enterprise intrinsic factors (trade unions, workers’ congresses) and the external environment (level of regional development), aiming to shed light on the exact reasons behind private enterprises’ non-compliance with their old-age insurance commitments. Considering that the phenomenon of private enterprises avoiding the purchase of old-age insurance is more serious in China, it is well represented. Therefore, this study critically examines this framework using data from 3,123 private enterprises collected in the 10th China Private Enterprise Survey (CPES).^1^ The purpose of this study is to shed light on actionable insights that government entities should consider when addressing this issue. This, in turn, will assist government agencies in formulating effective policies and provide a foundational reference for future academic research endeavors.

## 2 Literature review and theoretical hypotheses

The obligation of enterprises to engage in old-age insurance is subject to a multitude of influential elements. Since the 1960s, scholars across various nations have embarked on the examination of factors that impact participation in old-age insurance, predominantly concentrating on macro-level determinants. Noteworthy among these factors are macroeconomic variables encompassing the growth rate of the economy, income levels, price dynamics, currency stability, and prevailing interest rates. Additionally, legal and regulatory frameworks, the structure of property rights, the quality of regulatory mechanisms, and the efficacy of corruption control measures, along with demographic variables like population size, educational attainment, life expectancy, and urbanization patterns, have attracted scholarly attention (12–15). In the progression of research, an increasing number of scholars posit that an exploration into the factors influencing enterprises’ adherence to their responsibility of participating in old-age insurance from an enterprise-specific micro-level perspective would complement the existing body of research and yield a more pragmatic outlook (16). Consequently, certain scholars have undertaken pertinent investigations from the vantage points of both external and internal environmental determinants concerning participation in old-age insurance (17).

### 2.1 Relationship between enterprise profit and the basic old-age insurance coverage rate

Since private enterprises are usually owned or invested by individuals, their profitability is closely related to the economic returns of the operators (18). As a result, the profit-centered nature of private enterprises has prompted them to explore various avenues to reduce employee-related costs (19). While employee compensation packages in private enterprises typically fall behind those in foreign and state-owned enterprises, the disparity is not extensive. As a result, certain private enterprises may adopt the strategy of reducing or completely exempting themselves from social security contributions, including old-age insurance, when the economic situation is unfavorable. This is usually achieved by reducing the benefits given to employees, all to optimize the earnings of the enterprise owner (20). Further, in less profitable enterprises, their employees tend to be less skilled on average, which means that their pay packages will also be lower than in more profitable enterprises (21). In addition, employees with lower skill levels tend to be less educated as well, which may result in a lack of awareness regarding pension participation (22). This leads to a low willingness to participate in old-age insurance in less profitable enterprises, both among the managers and among the employees. Building upon the aforementioned body of research, the present study posits the hypothesis that a negative correlation exists between the profitability of private enterprises and the coverage rate of fundamental old-age insurance. The hypothesis is formulated as follows:

*H1*: Lower enterprise profitability is associated with a decreased coverage rate of basic old-age insurance.

### 2.2 Relationship between the regional development level and the basic old-age insurance coverage rate

Drawing from the unique context and developmental strategy of China, a notable divergence in economic progress across its regions significantly influences the commitment of enterprises to participate in old-age insurance (23). Shaped by the dynamics of reform and opening up, the eastern region of China leverages its geographic advantage, political framework, and an array of sociopolitical factors to prioritize and expedite development. Conversely, the central and western regions, situated inland, experience a more gradual pace of reform and opening up, coupled with comparatively lagging economic advancement. This dichotomy in economic development levels underscores the gradient disparities among China’s eastern, central, and western regions. The repercussions of this regional economic variance reverberate through two key dimensions concerning employee old-age insurance coverage. Firstly, the incongruity in regional economic progress translates into divergences in employee incomes in enterprises. The economically disadvantaged locales witness diminished staff incomes, thereby fostering a reluctance to participate in old-age insurance due to lower financial capacity, ultimately leading to reduced rates of insurance engagement. Secondly, the dissimilarities in regional economic growth give rise to disparate fiscal conditions for local governments, consequently yielding varying degrees of financial subsidies or policy offerings for old-age insurance (24). Consequently, regions endowed with greater affluence extend more comprehensive subsidies, engendering higher coverage rates, while financially constrained regions, burdened by limited fiscal resources or substantial historical debts, manifest lower participation rates. Additionally, the regional developmental gradient manifests an influence over the compliance of private enterprises with their obligation to participate in old-age insurance, specifically in terms of systemic implementation (25). Enterprises are incentivized to sidestep their responsibilities concerning employee social insurance participation through unscrupulous practices, particularly conspicuous in China’s intricate and expansive governmental structures. This phenomenon is often fueled by misinterpretations of social insurance obligations, conflicting interests, and ambiguous definitional boundaries, thereby catalyzing rent-seeking behaviors (26). Notably, policy execution exhibits noteworthy disparities across disparate Chinese regions. Regions with robust economic advancement typically exhibit more robust regulatory endeavors and well-entrenched frameworks, consequently fostering enhanced adherence to policy implementations (27). Moreover, heightened legal awareness, robust legal enforcement capabilities, and stringent punitive measures characterize developed regions. Enterprises situated in these locales consequently face elevated costs when endeavoring to evade employee insurance-related responsibilities (28). In summation, this paper advances the following assertion:

*H2*: The degree of regional development exhibits a positive correlation with the coverage rate of fundamental old-age insurance.

### 2.3 Relationship between labor union and workers’ congress and the basic old-age insurance coverage rate

Supporting and defending the legitimate rights and interests of workers is both a social and historical obligation of trade unions and workers’ congresses (29). Trade unions and workers’ congresses are governance mechanisms for improving employee welfare and have played an important role in promoting pension participation in private enterprises (30). Through negotiation and consultation with enterprise management, labor unions, and workers’ congresses can push employers to improve employee benefits, including pension insurance, health insurance, and paid leave (31). In addition, labor unions and workers’ congresses are involved in the development and promotion of the company’s welfare policies to ensure that the needs of the employees are adequately taken into account. Trade unions and workers’ congresses are also able to offer collective welfare programs that provide more favorable insurance and welfare conditions for members (32). Through training and educational activities, unions and workers’ congresses raise employees’ awareness of benefit programs so that they can better enjoy the benefits offered by the company (33). Therefore, the establishment of labor unions and workers’ congresses has a significant effect on the coverage of employees’ insurance (34). In summary, the following hypotheses are proposed in this paper:

*H3*a: The existence of a labor union correlates positively with the coverage rate of basic old-age insurance.

*H3*b: The presence of a workers' congress correlates positively with the coverage rate of basic old-age insurance.

### 2.4 The moderating role of regional development level on the relationship between enterprise profit and basic old-age insurance coverage rate

The participation of private enterprises in the old-age insurance sector is mainly influenced by their profitability, while the level of regional development plays a moderating role in this dynamic. Regions with strong economic growth tend to have more robust institutional frameworks and more efficient policy implementation (35). Moreover, in developed regions, government oversight of private enterprises has a higher degree of authority and impartiality, ensuring that they take their pension-related obligations seriously (36). This unbiased supervision is not affected by differences in enterprise profitability. Thus, this fair and strict external environment helps to constrain and mitigate the impact of private enterprises’ profitability on their fulfillment of employee old-age insurance responsibilities. Combined with stricter supervision and better institutionalization, employees in economically developed regions show a higher awareness of their rights. This collective awareness helps to create an environment that is more conducive to safeguarding employees’ rights (37). Private enterprises that fail to fulfill their old-age insurance obligations are prone to employee dissatisfaction and increased employee turnover. The combination of these factors increases the costs borne by employers for failing to participate in employee old-age insurance programs. As a result, in developed regions, the extent to which the economic returns of private enterprises influence their fulfillment of employee old-age insurance obligations is significantly reduced. However, the opposite is true in economically disadvantaged regions. Employees in these regions have relatively weak awareness of their rights and lack a sound grievance mechanism; they can only acquiesce to their employers’ old-age insurance practices or choose to resign (38). The relatively low level of economic development in these regions exacerbates the challenge of finding alternative employment or negotiating improved pension benefits. As a result, private enterprises operating in these regions have greater discretion in deciding whether or not to participate in employee old-age insurance programs (39). These firms exercise greater autonomy in deciding whether to provide insurance benefits to their employees based on their financial situation. Therefore, in economically underdeveloped regions, private enterprises are more likely to be affected by economic factors in fulfilling their obligation to participate in old-age insurance. In summary, the following hypotheses are formulated:

*H4*: The level of regional development plays a negative moderating in the relationship between enterprise profitability and the coverage rate of basic old-age insurance.

### 2.5 The moderating role of labor union presence and workers’ congress on the relationship between enterprise profit and basic old-age insurance coverage rate

The ability of private enterprises to fulfill their commitment to participate in old-age insurance depends on their profitability, and this link also depends on the presence of trade unions and staff councils in private enterprises. Trade unions and workers’ congresses are indispensable institutions for balancing the interests of employees and investors and tend to ensure increased employee pension insurance coverage (40), especially when private enterprises tend to reduce their pension insurance obligations as a result of declining profitability (41). Employee organizations, including trade unions and workers’ congresses, have strong influence, so they have an incentive to fight for more insurance provisions for their constituents and to engage in politically motivated negotiations with employers (42), which in turn increase pension insurance coverage of private enterprises (43). Thus, it is not only in better-run enterprises, but even in less prosperous enterprises, the presence of labor unions improves employees’ pension benefits and coverage. To summarize, this paper presents the following hypotheses:

*H5*a: In private enterprises with a labor union, the coverage rate of basic old-age insurance is relatively less impacted by enterprise profit compared to enterprises devoid of a labor union.

*H5*b: In private enterprises with a workers' congress, the coverage rate of basic old-age insurance is relatively less influenced by enterprise profit in contrast to enterprises without a workers' congress.

**Figure 1:**
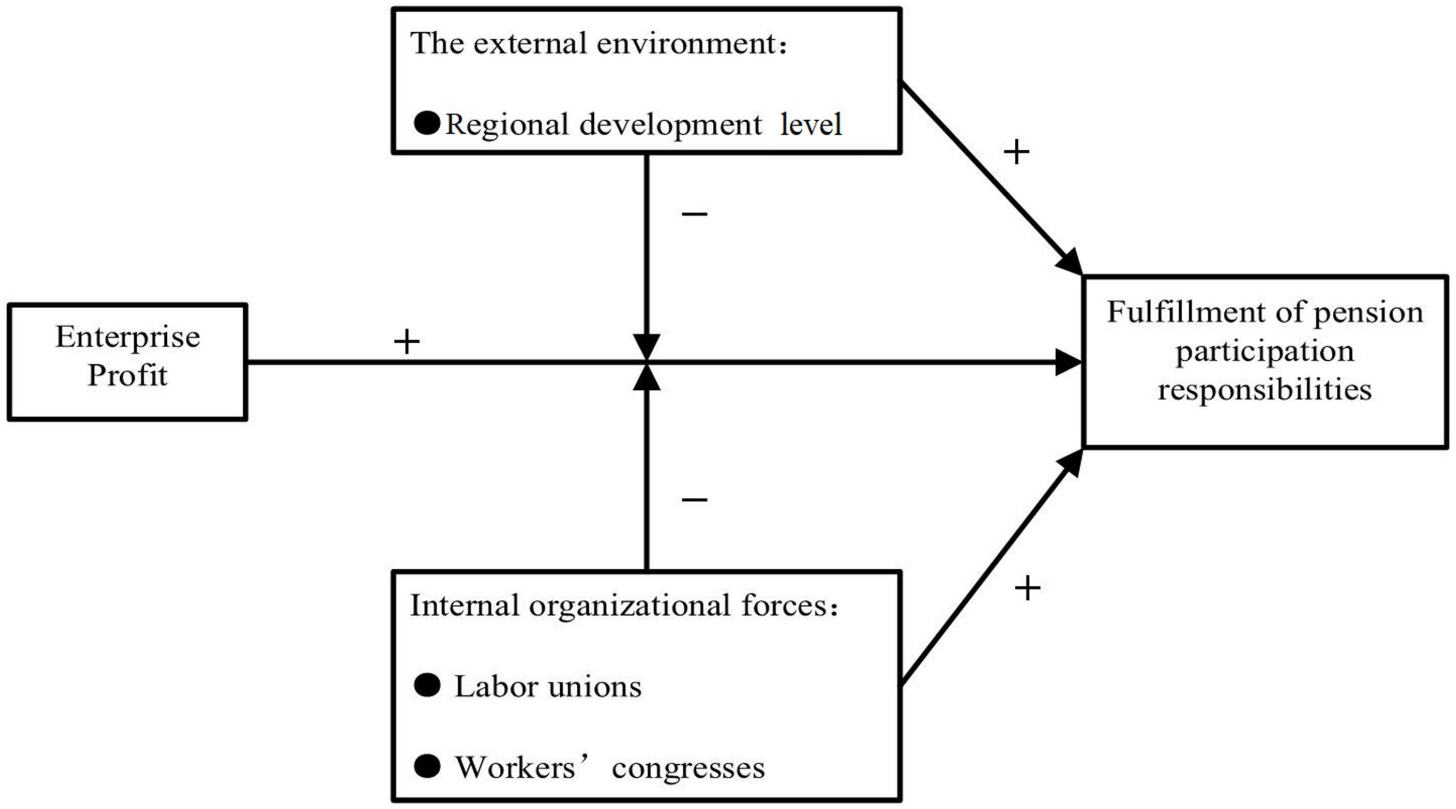
Research Framework.

This study undertakes an analysis of the determinants that shape the employee old-age insurance coverage rate at the enterprise level. Initially, a primary rationale behind enterprises failing to meet the responsibility of furnishing employees with social insurance lies in financial considerations. Social insurance premiums constitute a substantial fraction of an enterprise’s labor-related expenditures, and an elevated contribution rate for old-age insurance further magnify the social security payment burden of Chinese enterprises (44). Subsequently, viewed through the lens of regional disparities, the developmental status of the region wherein the enterprise is situated emerges as a pivotal indicator of the local economic milieu and the potency of governmental oversight. Notably, the regional development level stands as a pivotal factor influencing enterprises’ fidelity to old-age insurance participation, underpinned by divergences in policy implementation across regions. Additionally, the influential roles occupied by labor unions and workers’ congresses extend to safeguarding the fundamental rights and interests of workers while harmonizing the competing interests of employees and investors (9). This paper reviews the development of the urban and rural old-age security systems in China, and discusses the challenges in the process of constructing a sustained old-age security system. Although funding gaps and empty individual accounts have imposed a heavy burden on the sustainability of China’s urban pension system, there is a relatively high coverage rate of 35.3 percent for urban workers. However, China’s pension system provides a low coverage rate for rural farmers. The more rapidly aging population and lower incomes in rural areas pose challenges to the vulnerable rural household support system. The separation of the old age security system between rural and urban areas also puts great pressure as a result of urbanization on farmers who lose their farmland. Therefore, China must speed up the reform of its old-age security system to provide institutional support for its economic and social transition (45). Thus, this study undertakes an exploration of the interplay between enterprise profitability, the external context (regional development), and internal organizational dynamics (labor union and workers’ congress) that collectively shape private enterprises’ participation in old-age insurance. The schematic representation of the research framework is provided in Figure 1.

## 3 Research design and analysis

### 3.1 Data

The empirical data employed in this research are derived from the 10th CPES (Chinese Private Enterprise Survey), a collaborative initiative involving the Central United Front Work Department, the All-China Federation of Industry and Commerce, the State Administration for Industry and Commerce, and the China Association of Private Economy in 2012. The survey design embraced a multi-stage stratified random, targeting different industries and different scale private enterprises spanning 31 provinces, municipalities, and autonomous regions in China, which was with strong representativeness. From this survey, 5,073 valid responses were garnered, and subjected to further meticulous screening processes. This entailed the removal of samples marked by missing crucial variables and instances wherein old-age insurance coverage rates exceeded 100% (2, 3). Moreover, to temper the influence of outliers, a bilateral truncation technique was applied to two continuous variables: enterprise age and enterprise profit. After undergoing these rigorous selection procedures, the study ultimately harnessed data derived from 3,123 sampled enterprises.

### 3.2 Variable definitions

#### 3.2.1 Employee old-age insurance coverage rate

In this study, the employee old-age insurance coverage rate serves as a metric to gage the extent of an enterprise’s commitment to providing old-age insurance for its employees. This rate signifies the proportion of individuals effectively covered by insurance relative to the total count of company employees. Existing research on insurance coverage rates predominantly concentrates on macro-level assessments, drawing from data encompassing regional coverage rates on a broader scale, thereby often neglecting the nuanced impact of micro-level variables on private enterprises (45). This study, however, takes a granular approach by scrutinizing the micro-level dynamics within companies. The coverage rate is computed by dividing the number of employees under old-age insurance coverage by the overall workforce count.

#### 3.2.2 Enterprise profit

This study adopts enterprise net profit to measure its profitability. Net profit denotes the retained earnings of a company after the prescribed payment of income taxes in the total profit, serving as a primary indicator to measure an enterprise’s operational performance and a foundational tool for assessing its profitability.

#### 3.2.3 Regional development level

Taking into account the economic development and geographical location of diverse provinces and cities in mainland China, this study categorizes the mainland into three principal regions: East, Central, and West. The Eastern region encompasses Hainan, Guangdong, Shandong, Fujian, Zhejiang, Jiangsu, Shanghai, Liaoning, Hebei, Tianjin, and Beijing. The Central region encompasses Hunan, Hubei, Henan, Jiangxi, Anhui, Heilongjiang, Jilin, and Shanxi. The Western region encompasses Xinjiang, Qinghai, Gansu, Ningxia, Shaanxi, Tibet, Yunnan, Guizhou, Chongqing, Sichuan, Guangxi, and Inner Mongolia. In this study, each region’s development level is assigned a numeric value, with the West designated as 1, the Central as 2, and the East as 3.

#### 3.2.4 Labor union and workers’ congress

Labor unions and workers’ congresses constitute employee-centric organizations within a company that actively champion employee benefits, often presenting a counterbalancing influence against the interests of the company’s investors. This study separately examines the impact of establishing labor unions and workers’ congresses in private enterprises on fulfilling obligations of offering old-age insurance. In this context, “1” is assigned to signify the establishment of a labor union or workers’ congress, while “0” denotes the absence of such establishment.

#### 3.2.5 Control variables

To elucidate the theoretical hypotheses of this study with greater clarity, the analysis incorporates control variables encompassing enterprise age and enterprise size. Enterprises with a lengthier operational history generally cultivate a more favorable public and official perception. Typically, these enterprises exhibit a heightened propensity to uphold a robust employee insurance coverage rate, driven by the imperative to maintain a positive corporate image. This, in turn, significantly impacts the enterprise’s adherence to obligations concerning employee old-age insurance. Thus, the study controls over enterprise age, quantified as the difference between the survey year and the year of establishment. Furthermore, larger enterprises frequently wield a more pronounced influence over local employment dynamics, which consequently attracts greater attention and reliance from local governmental bodies. In this context, local authorities might demonstrate a more tempered approach to penalizing larger entities for non-compliance with insurance coverage, subsequently influencing the strategic choices and behavioral patterns of these companies concerning employee old-age insurance (46). Consequently, the study also controls over enterprise size, categorizing it into four tiers based on the workforce magnitude: 1 represents enterprises with fewer than 100 employees, 2 denotes those with 100 to 499 employees, 3 signifies the range of 500 to 1,000 employees, and 4 designates enterprises employing over 1,000 personnel. A comprehensive elucidation of each variable is available in Table 1.

**Table 1:** Measurement of variables.

Variable	Variable name	Variable description
Dependent variable	Employee old-age insurance coverage rate	The number of employees purchasing old-age insurance in the company divided by the total number of company employees
Independent variable	Enterprise profit	Net profit of the enterprise (Ten million yuan)
Regulated variable	Regional development level	Western = 1, Central = 2, Eastern = 3
	Labor union	Established = 1, Not established = 0
	Workers’ congress	Established = 1, Not established = 0
Control variable	Enterprise age	Subtract the year of establishment from the survey year (2010)
	Enterprise size	Number of employees: <100 = 1, 100 ~ 499 = 2,500 ~ 1,000 = 3, > 1,000 = 4

### 3.3 Descriptive statistics

Table 2 illustrates the distribution of fundamental characteristics inherent to the sampled enterprises. This survey encompasses an expansive spectrum of enterprise magnitudes, with the preponderance constituting entities employing fewer than 100 personnel, aligning with the prevalent landscape of small-scale private enterprises in China. The surveyed enterprises span diverse years of registration, encompassing both recently established enterprises and well-established firms. The survey encompasses a gamut of enterprise types, including sole proprietorships, partnerships, private limited companies, and joint-stock companies, effectively capturing the regional diversity encapsulated within the eastern, central, and western domains. Given the historical proliferation of private enterprises in the eastern region, a greater concentration of enterprises from this locale is observed, an observation that mirrors the overarching distribution pattern of private enterprises across China. The comprehensive and representative nature of the sample data augments the study’s robustness and persuasiveness.

**Table 2:** Descriptive analysis of the samples.

Variables		Obs	Proportion (%)
Enterprise size	Less than 100 employees	1,960	62.76
	100 ~ 499 employees	903	28.91
	500 ~ 1,000 employees	151	4.84
	More than 1,000 employees	109	3.49
Registration year	Before 1994	248	7.94
	1995 ~ 1999	596	19.09
	2000 ~ 2004	1,015	32.50
	Since 2005	1,264	40.47
Enterprise type	Sole Proprietorship	315	10.09
	Partnership	110	3.52
	Private Limited Company	2,416	77.36
	Joint-stock Company	216	6.92
Region	Western	618	19.79
	Central	727	23.28
	Eastern	1,778	56.93

There are 66 missing values in the variable enterprise type.

### 3.4 Model construction

To investigate the effect of enterprise profit on enterprise basic old-age insurance responsibilities, we establish the following in Equation 1:


(1) $$Insurance_{i,2012}=\alpha_{0}+a{\mathrm{Profit}}_{i,2012}+dControls_{i,2012}+\varepsilon$$

Here, i denotes company, 2012 denotes the year, $\;Insurance_{i,2012}$ denotes the performance of company i in 2012. $Profit_{i,2012}$ denotes the profit of enterprise i in 2012, $Controls_{i,2012}$ denotes a random variable, and ε is a random disturbance term.

To further explore the moderating effect of labor unions, workers’ congress, and the level of regional development, we establish the following in Equations 2^4^:


(2) $$\begin{array}{l} Insurance_{i,2012}=\beta_{0}+\beta_{1}Profit_{i,2012}+\beta_{2}Union_{i,2012}\\ +\beta_{3}Profit_{i,2012}\times Union_{i,2012}+\beta_{4}Controls_{i,2012}+\varepsilon \end{array}$$

(3) $$\begin{array}{l} Insurance_{i,2012}=\gamma_{0}+\gamma_{1}Profit_{i,2012}+\gamma_{2}Congress_{i,2012}\\ +\gamma_{3}Profit_{i,2012}\times Congress_{i,2012}+\gamma_{4}Controls_{i,2012}+\varepsilon \end{array}$$

(4) $$\begin{array}{l} Insurance_{i,2012}=\delta_{0}+\delta_{1}{\mathrm{Profit}}_{i,2012}+\delta_{2}Region_{i,2012}\\ +\delta_{3}Profit_{i,2012}\times Region_{i,2012}+\delta_{4}Controls_{i,2012}+\varepsilon \end{array}$$

Here, $\;Union_{i,2012}$ denotes the establishment of labor union of company i in 2012, $Congress_{i,2012}$ denotes the establishment of workers’ congress of company i in 2012, $Region_{i,2012}$ indicates the economic development of the region where company i is located in 2012. $Controls_{i,2012}$ denotes a random variable, and ε is a random disturbance term.

## 4 Results

### 4.1 Correlation analysis

Drawing upon the dataset from the Chinese Private Enterprise Survey, this study has systematically examined and statistically analyzed the data pertaining to the rate of old-age insurance coverage, as meticulously outlined in Table 3. As delineated in the tabulated data, a mere 627 out of the entire cross-section of private enterprises sampled (20.08%) have demonstrated comprehensive adherence to the stipulated requirement by enrolling all their employees in the old-age insurance program. Conversely, a substantial contingent of enterprises, constituting a total of 1,683 cases (53.89% of the total sample), exhibit a considerably diminished extent of compliance, with fewer than half of their employees encompassed within the purview of old-age insurance. Intriguingly, an additional stratum is unveiled wherein enterprises encompassing less than a quarter of their workforce within the scope of old-age insurance coverage, while making up 42.59% of the entire sample, underscoring the prevailing disjunction between regulatory expectations and the actual implementation by private enterprises.

**Table 3:** Old-age insurance coverage rate in private enterprise.

Old-age insurance coverage rate	Obs	Proportion (%)	Cumulative sample size	Cumulative proportion (%)
Coverage rate<25%	1,330	42.59	1,330	42.59
25% ≤ Coverage rate<50%	353	11.30	1,683	53.89
50% ≤ Coverage rate<75%	427	13.67	2,110	67.56
75% ≤ Coverage rate<100%	386	12.36	2,496	79.92
Coverage rate = 100%	627	20.08	3,123	100

Moving forward, Table 4 serves as a repository presenting the computed mean, standard deviation, and Pearson correlation coefficient matrix encapsulating the variables subject to investigation in this study. A detailed perusal of the correlation coefficients enshrined in Table 4 reveals that the rate of old-age insurance coverage within private enterprises exhibits a statistically significant positive correlation with variables including enterprise age, profitability, regional developmental status, and the establishment of either a labor union or a workers’ congress. These emergent correlations bear a striking alignment with the underlying theoretical expectations, thereby furnishing preliminary substantiation for several of the hypotheses advanced in this study. However, the attainment of robust and comprehensive conclusions necessitates a deeper level of statistical scrutiny, to be achieved through the deployment of regression analysis techniques.

**Table 4:** Mean, standard deviation, and correlation coefficients of the variables.

Variables	Mean	SD	1	2	3	4	5	6
1. Coverage rate	0.445	0.403	1					
2. Enterprise size	1.491	0.746	0.151^***^	1				
3. Enterprise age	9.266	5.292	0.251^***^	0.315^***^	1			
4. Enterprise profit	0.447	1.324	0.240^***^	0.474^***^	0.184^***^	1		
5. Regional development level	2.371	0.793	0.171^***^	0.052^***^	0.161^***^	0.052^***^	1	
6. Labor union	0.497	0.500	0.281^***^	0.404^***^	0.353^***^	0.227^***^	0.080^***^	1
7. Workers’ congress	0.319	0.466	0.172^***^	0.306^***^	0.211^***^	0.174^***^	0.037^**^	0.447^***^

*p < 0.1, **p < 0.05, ***p < 0.001.

### 4.2 Regression analysis

When the multiple regression model is used to evaluate the causal relationship, there may be a certain linear relationship between the explanatory variables, and the parameter β will not be effectively identified. Multicollinearity is generally diagnosed by variance inflation factor and tolerance, which are reciprocals of each other. Table 5 reports the VIF and tolerance of each variable. Since the maximum VIF is 1.53, less than the rule-of-thumb value of 10, it means that there is no severe collinearity problem between the explanatory variables.

**Table 5:** The variance inflation factor of each variable.

Variables	VIF	Tolerance
Enterprise age	1.22	0.822
Enterprise size	1.53	0.652
Enterprise profit	1.29	0.772
Regional development level	1.03	0.973
Labor union	1.47	0.682
Workers’ congress	1.28	0.780

To scrutinize the hypotheses posited in this study, a meticulous stepwise multiple regression analysis was undertaken to assess the impact of different factors on the extent of old-age insurance coverage within private enterprises. To initiate this analysis, firstly, control variables and the independent variable were introduced to discern their influence on the rate of employee old-age insurance coverage (Model 1). Following this, moderating variables, including the regional development level, the presence of labor unions, and the establishment of workers’ congresses, were introduced (Model 2). Consequently, Model 2 encapsulates the comprehensive effect, entailing control, independent, and moderating variables. In Model 3 to Model 5, three distinct interaction terms are sequentially introduced based on Model 2. To mitigate potential multicollinearity stemming from the introduction of interaction terms, a standardization procedure was initially employed for both independent and moderating variables before the computation of the interaction terms. Furthermore, to address multicollinearity concerns inherent in the interaction terms themselves, an established scholarly approach was adopted, involving the incremental introduction of each interaction term into the main effect model – a methodology proven effective in navigating such concerns. The results of the stepwise multiple regression analysis are meticulously delineated in Table 6.

**Table 6:** Regression analysis results.

Variables	Model 1	Model 2	Model 3	Model 4	Model 5
*Control variables*					
Constant	0.276^***^ (15.34)	0.145^***^ (5.70)	0.133^***^ (5.10)	0.144^***^ (5.68)	0.144^***^ (5.69)
Enterprise size	−0.009 (−0.79)	−0.045^***^ (−4.12)	−0.047^***^ (−4.23)	−0.047^***^ (−4.24)	−0.045^***^ (−4.09)
Enterprise age	0.017^***^ (12.16)	0.011^***^ (7.97)	0.011^***^ (8.05)	0.011^***^ (7.77)	0.011^***^ (7.93)
*Independent variables*					
Enterprise profit	0.063^***^ (10.74)	0.060^***^ (10.43)	0.095^***^ (5.33)	0.097^***^ (6.42)	0.066^***^ (8.08)
*Moderating variables*					
Regional development level		0.064^***^ (7.53)	0.063^***^ (7.44)	0.063^***^ (7.45)	0.063^***^ (7.47)
Labor union		0.153^***^ (9.51)	0.153^***^ (9.51)	0.144^***^ (8.81)	0.152^***^ (9.43)
Workers’ congress		0.037^**^ (2.31)	0.036^**^ (2.21)	0.039^**^ (2.42)	0.038^**^ (2.38)
*Interaction variables*					
Enterprise profit × Regional development level			−0.014^**^ (−2.10)		
Enterprise profit × Labor union				−0.042^***^ (−2.67)	
Enterprise profit × Workers’ congress					−0.011 (−1.06)
*R* ^2^	0.102	0.153	0.154	0.155	0.153
Adj. *R*^2^	0.110	0.151	0.152	0.153	0.151
*F*-value	117.84^***^	93.68^***^	81.02^***^	81.48^***^	80.46^***^

*p < 0.1, **p < 0.05, ***p < 0.001.

An examination of Model 1 presented in Table 6 underscores the substantial explanatory prowess of enterprise age and profit concerning variations in the old-age insurance coverage rate. The analysis demonstrates a statistically significant positive influence of enterprise profit on the fulfillment of employee old-age insurance obligations (β = 0.063, *p* < 0.01), thus substantiating hypothesis H1.

Progressing to Model 2, which introduces variables of regional development level, labor unions, and workers’ congresses while concurrently controlling for enterprise age, size, and profits, the model’s explanatory capacity is further expand. Notably, the analysis reveals a statistically significant positive impact of regional development level on the fulfillment of employee old-age insurance obligations (β = 0.064, *p* < 0.01), in line with hypothesis H2. Furthermore, the presence of a labor union demonstrates a pronounced positive effect on the fulfillment of employee old-age insurance obligations (β = 0.153, *p* < 0.01), providing empirical support for hypothesis H3a. Similarly, the existence of workers’ congresses demonstrates a noteworthy positive impact on the realization of employee old-age insurance obligations (β = 0.037, *p* < 0.05), thereby buttressing hypothesis H3b.

Proceeding to Model 3, which incorporates the interaction term between regional development level and enterprise profit, the analysis highlights a significant negative influence of this interaction on the fulfillment of employee old-age insurance obligations (β = −0.014, p < 0.05), congruent with hypothesis H4.

Analogously, Model 4 introduces the interaction term between labor unions and enterprise profit, underscoring a substantial negative effect of this interaction on the realization of employee old-age insurance obligations (β = −0.042, *p* < 0.01), consequently corroborating hypothesis H5a.

Lastly, Model 5 integrates the interaction term between workers’ congresses and enterprise profit, yet the results reveal the absence of a significant impact on the fulfillment of employee old-age insurance obligations (β = −0.011, *p* > 0.1), consequently failing to validate hypothesis H5b.

**Figure 2:**
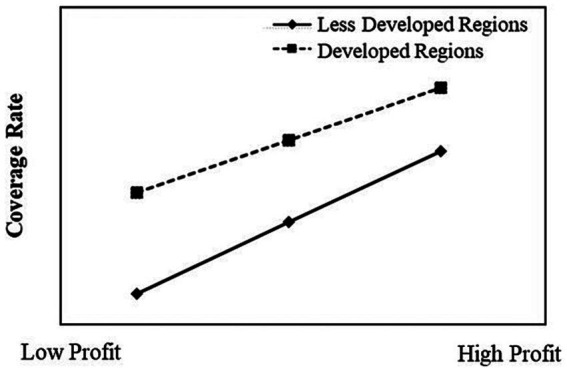
The moderating effect of Regional Development Level.

**Figure 3:**
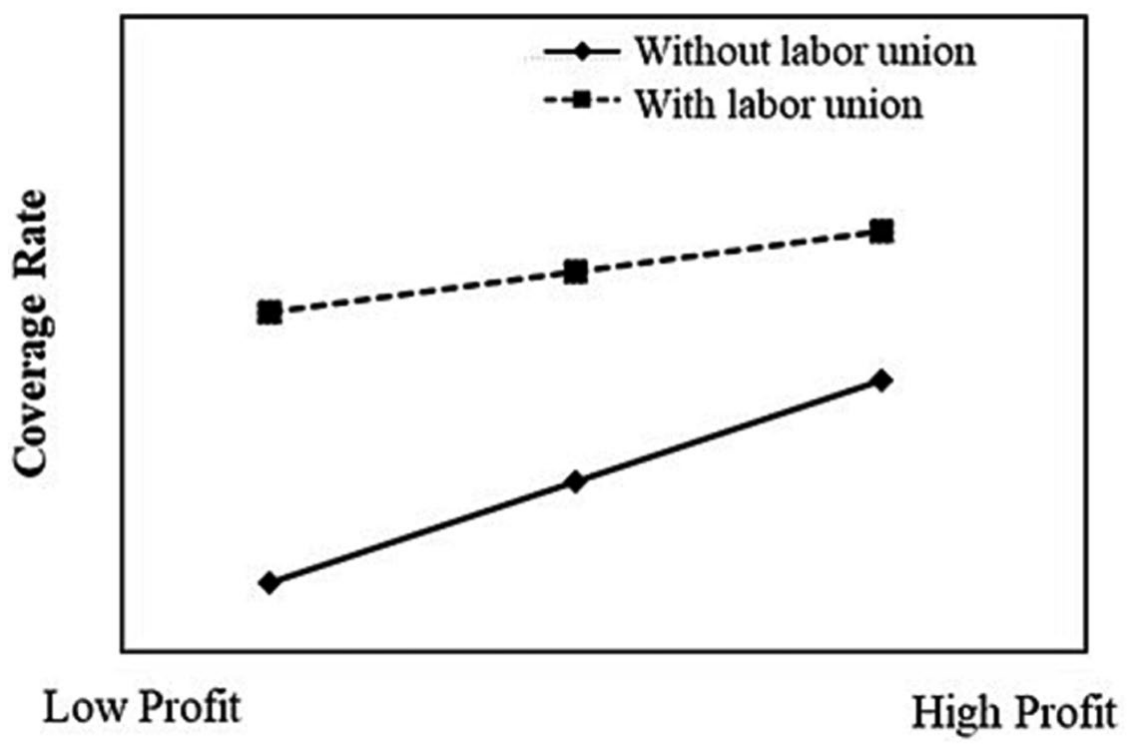
The moderating effect of Labor Union.

This study portrays the moderation effect plots in Figures 2, 3, which effectively elucidate the interplay between regional development level, labor unions, and their interactive effects on the intricate relationship that exists between enterprise profit and the rate of old-age insurance coverage.

Figure 2 visually depicts the negative moderation effect instigated by the regional development level on the nexus between enterprise profit and the old-age insurance coverage rate. Conspicuously, enterprises positioned within more developed regions, influenced by an amalgamation of environmental determinants including regulatory dynamics inherent to the regional milieu, manifest a relatively heightened old-age insurance coverage rate. Moreover, the previously discerned positive correlation between enterprise profit and coverage rate tends to exhibit a more tempered manifestation in these enterprises. Conversely, enterprises nestled in regions characterized by a lower level of developmental advancement, owing to the presence of a comparatively permissive regulatory backdrop, evince a heightened susceptibility to the influences of enterprise profit. Consequently, these entities display a more pronounced positive correlation between enterprise profit and the rate of coverage.

Figure 3 serves to illustrate the negative moderation effect orchestrated by the presence of labor unions on the intricate relationship between enterprise profit and the old-age insurance coverage rate. Within the domain of enterprises equipped with labor unions, the impact of enterprise profit upon the coverage rate assumes a relatively diminished magnitude. Conversely, in the absence of labor unions, the sway wielded by enterprise profit upon the participation rate acquires a more substantial character, thereby engendering an augmented positive correlation.

### 4.3 Robustness check

Through the implementation of a variable substitution approach, we have chosen to replace the central independent variable that plays a crucial role in our study. Drawing upon the viewpoints presented by AO Trostel and other respected scholars (47), it becomes apparent that private enterprises attach elevated importance to the expansion of their business operations and the scalability of their sales. Notably, the enterprises under examination possess relatively concise operational histories, which can potentially lead to variations in their financial systems and statements when compared to publicly listed enterprises that adhere to more traditional practices. As a result of these considerations, we have decided to replace the primary independent variable, which was originally enterprise profit, with the metric of the company’s operational revenue. This new metric serves as an indicator of organizational performance. The outcomes arising from this substitution are meticulously detailed in Table 7. In Model 6, it becomes distinctly evident that the operational revenue of the company is conspicuously and positively correlated with the extent of coverage provided by the old-age insurance, reaching a remarkable level of significance at 1%. Continuing Model 6 through 9, the data brings to light that the interaction term involving operational revenue and the regional level of development, as well as operational revenue and the presence of a labor union, sustain notably significant and negative correlations. These correlations hold a level of significance of 5%. This specific set of findings underscores that even after the adjustment in the methodology used for measuring enterprise profit, the fundamental conclusions derived within the scope of this study exhibit resilience and robustness.

**Table 7:** Results of core explanatory variable substitution test.

Variables	Model 6	Model 7	Model 8	Model 9
Operating revenue	0.004^***^ (8.65)	0.006^***^ (4.58)	0.007^***^ (4.65)	0.004^***^ (5.52)
Regional development level		0.061^***^ (6.62)		
Labor union			0.115^***^ (5.57)	
Workers’ congress				0.041^**^ (2.41)
Operating revenue × Regional development level		−0.001^**^ (−1.99)		
Operating revenue × Labor union			−0.004^**^ (−2.38)	
Operating revenue × Workers’ congress				−0.001 (−1.13)
Control variables	Yes	Yes	Yes	Yes
*R* ^2^	0.086	0.138	0.139	0.137
Adj. *R*^2^	0.086	0.136	0.137	0.135
*F*-value	92.72^***^	67.28^***^	67.57^***^	66.84^***^

*p < 0.1, **p < 0.05, ***p < 0.001.

Recognizing the potential introduction of estimation bias attributed to the omission of pertinent variables, this study has undertaken a comprehensive robustness assessment through the introduction of control variables. Prior scholarly endeavors have underscored the influence exerted by entrepreneurs’ characteristics upon a company’s engagement in social responsibility activities (48). As such, to comprehensively address potential confounding factors, this analysis incorporates four control variables that encapsulate entrepreneurs’ attributes. These variables encompass age, gender, educational attainment, and affiliation with political entities such as the Communist Party of China or democratic parties. Within the initial regression model, the variable of party membership is treated as a binary indicator, assuming a value of 1 in instances where the entrepreneur is affiliated with either the National People’s Congress or the Chinese People’s Political Consultative Conference, and a value of 0 otherwise. The detailed findings stemming from this analysis are meticulously documented in Table 8. Upon incorporation of the control variables, the regression coefficients associated with enterprise profit, regional development level, the presence of labor unions, and the establishment of workers’ congresses persistently sustain significant and positive correlations with the rate of old-age insurance coverage. Furthermore, we again validated the significance of the interaction terms, affirming the robustness of the conclusions drawn in this study.

**Table 8:** Results of analysis with added control variables.

Variables	Model 10	Model 11	Model 12	Model 13	Model 14
*Control variables*					
Constant	−0.049 (−0.87)	−0.163^***^ (−2.77)	−0.174^***^ (−2.95)	−0.164^***^ (−2.78)	−0.163^***^ (−2.77)
Enterprise size	−0.051^***^ (−4.46)	−0.076^***^ (−6.60)	−0.079^***^ (−6.82)	−0.077^***^ (−6.71)	−0.076^***^ (−6.60)
Enterprise age	0.014^***^ (9.83)	0.010^***^ (6.89)	0.010^***^ (6.99)	0.010^***^ (6.61)	0.010^***^ (6.90)
Gender	−0.037^*^ (−1.87)	−0.023 (−1.19)	−0.023 (−1.21)	−0.023 (−1.19)	−0.023 (−1.20)
Education	0.070^***^ (10.40)	0.066^***^ (10.13)	0.067^***^ (10.15)	0.066^***^ (10.10)	0.067^***^ (10.14)
Age	0.003^***^ (3.60)	0.003^***^ (3.07)	0.003^***^ (2.97)	0.003^***^ (3.05)	0.003^***^ (3.07)
Party member	0.044^***^ (2.91)	0.023 (1.52)	0.023 (1.54)	0.022 (1.50)	0.023 (1.53)
*Independent variable*					
Enterprise profit	0.109^***^ (11.43)	0.098^***^ (10.47)	0.167^***^ (5.03)	0.166^***^ (6.35)	0.095^***^ (6.82)
*Moderating variable*					
Regional development level		0.063^***^ (7.34)	0.059^***^ (6.69)	0.062^***^ (7.24)	0.063^***^ (7.34)
Labor union		0.129^***^ (7.83)	0.129^***^ (7.84)	0.109^***^ (6.11)	0.130^***^ (7.83)
Workers’ congress		0.030^*^ (1.80)	0.027^*^ (1.66)	0.031^*^ (1.88)	0.030^*^ (1.81)
*Interaction variables*					
Enterprise profit × Regional development level			−0.026^**^ (−2.17)		
Enterprise profit × Labor union				−0.077^***^ (−2.80)	
Enterprise profit × Workers’ congress					0.006 (0.32)
*R* ^2^	0.153	0.192	0.194	0.194	0.192
Adj. *R*^2^	0.151	0.190	0.191	0.191	0.190
*F*-value	74.95^***^	68.88^***^	63.13^***^	63.48^***^	62.61^***^

*p < 0.1, **p < 0.05, ***p < 0.001.

Because this study is based on cross-sectional data, there may be heteroscedasticity problems. Therefore, we adopted the method of heteroscedasticity-robust standard errors for testing, which enables us to obtain reliable results even in unequal variances (49, 50). As shown in Table 9, the model still passed the significance test and demonstrated good robustness even after applying this method.

**Table 9:** Results of analysis with heteroscedasticity-robust standard errors applied.

Variables	Model 15	Model 16	Model 17	Model 18	Model 19
*Control variables*					
Constant	0.276^***^ (15.49)	0.145^***^ (5.86)	0.133^***^ (5.24)	0.144^***^ (5.84)	0.144^***^ (5.85)
Enterprise size	−0.009 (−0.81)	−0.045^***^ (−4.21)	−0.047^***^ (−4.31)	−0.047^***^ (−4.29)	−0.045^***^ (−4.17)
Enterprise age	0.017^***^ (12.32)	0.011^***^ (7.87)	0.011^***^ (7.94)	0.011^***^ (7.68)	0.011^***^ (7.83)
*Independent variables*					
Enterprise profit	0.063^***^ (11.68)	0.060^***^ (11.38)	0.095^***^ (7.71)	0.097^***^ (6.05)	0.066^***^ (9.62)
*Moderating variables*					
Regional development level		0.064^***^ (7.59)	0.063^***^ (7.53)	0.063^***^ (7.51)	0.063^***^ (7.52)
Labor union		0.153^***^ (9.38)	0.153^***^ (9.37)	0.144^***^ (8.74)	0.152^***^ (9.32)
Workers’ congress		0.037^**^ (2.37)	0.036^**^ (2.27)	0.039^**^ (2.48)	0.038^**^ (2.42)
*Interaction variables*					
Enterprise profit × Regional development level			−0.014^**^ (−2.75)		
Enterprise profit × Labor union				−0.042^***^ (−2.57)	
Enterprise profit × Workers’ congress					−0.011 (−1.17)
*R* ^2^	0.102	0.153	0.154	0.155	0.153
Adj. *R*^2^	0.101	0.151	0.152	0.153	0.151
*F*-value	130.99^***^	107.10^***^	103.21^***^	93.45^***^	94.44^***^

*p < 0.1, **p < 0.05, ***p < 0.001.

## 5 Discussion

The improvement of enterprise profit can effectively promote the old-age insurance participation. Historically, private enterprises in China have grappled with the exigencies of thriving in an intensely competitive landscape. Compared to other types of enterprises, their responsibility to participate in insurance imposes a heavier burden, thereby constricting the flow of enterprise funds and inhibiting their progression (51). In response, enterprises often resort to various strategies to evade contributions, such as concealing employees, underreporting wages, and employing other measures. Recent research indicates that reducing the burden of social security not only alleviates the strain on enterprises and promotes employment but also increase the enthusiasm of both enterprises and employees to participate in insurance. This, in turn, enhances the input–output dynamics of enterprises (51–53). Consequently, increasing enterprise profitability may lead to a higher coverage rate for employee basic old-age insurance.

The regional development level has a significant impact on old-age insurance participation. As documented in certain studies, a predominant portion of enterprises expresses a yearning for regulatory entities to prompt all peer enterprises to dutifully honor their social insurance commitments while concurrently fostering an impartial competitive environment (54). However, in different regions, and regional development levels, the supervision methods and administrations are also different. Many studies also show that the regional development level has a significant impact on the participation rate (55). The establishment of a robustly structured social insurance framework requires governmental enforcement, which presents formidable regulatory complexities for numerous administrations and stands as a pivotal concern for social insurance regulators in China.

The establishment of labor unions and workers’ congresses can improve old-age insurance participation. With the reform of the social security systems, the relationship between workers enterprises, and employers has undergone profound changes. Unlike the social security system based on state-owned enterprises in the past, the existing legal system is not sufficient to protect workers. Research has shown that labor unions and workers’ congresses are beneficial in protecting the rights and interests of private enterprise employees in participating in old-age insurance and supervising private enterprises to fulfill their obligations (56, 57). With the progress of society and the increasing awareness of workers’ rights protection, these employee organizations can play a significant role in safeguarding employee rights and coordinating labor-capital relations through improving organizational mechanisms.

In underdeveloped areas and private enterprises without labor unions, the impact of enterprise profits on old-age insurance participation is more pronounced. Underdeveloped areas lack sound appeal mechanisms, and in enterprises without established unions, employees have weak awareness of their rights and are forced to accept their employer’s old-age insurance arrangements or choose to resign (58, 59). Therefore, in this situation, private enterprises have greater autonomy in fulfilling their old-age insurance obligations, and the participation rate is more easily affected by enterprise profits (41).

This study have some limitations and suggestions for future research. First, we cannot entirely rule out the possibility that our findings could be affected by common method bias, as we tested our hypotheses using data collected from a single source and only selected cross-sectional data for analysis. Future research should consider studying from multiple data sources, using panel data, and addressing endogeneity issues to better understand the causal relationship. Second, this study mainly focuses on Chinese private enterprises. Due to the differences in ownership between private and other types of enterprises, such as state-owned enterprises, caution should be exercised when generalizing our findings to other types of enterprises. If we can compare the factors influencing the participation rate of old-age insurance for different types of enterprises, we believe that significant findings can be obtained.

## 6 Conclusion

This study constructs a comprehensive research framework, encompassing factors such as enterprise profitability, external environmental influences, and internal organizational dynamics to dissect the various factors that influence private enterprises’ adherence to their imperative commitment to engaging with the old-age insurance systems, while also delving into the intricate interplay among these factors. The study is grounded in empirical data extracted from the 10th National Survey of Private Enterprises, featuring a sample size of 3,123 enterprises. This robust dataset yielded significant research insights. Firstly, the fulfillment of old-age insurance participation by private enterprises is profoundly impacted by their profitability. Notably, as profits surge, the rate of employee participation proportionally escalates. Secondly, the engagement of private enterprises in the old-age insurance scheme is significantly affected by the external operational environment. Regions of higher development exhibit a corresponding elevation in insurance coverage rates. Thirdly, the obligation of private enterprises to partake in old-age insurance is notably influenced by the presence of labor unions. Enterprises that harbor labor unions tend to have higher coverage rates. Fourthly, the level of regional development manifests a paradoxical effect on the relationship between enterprise profits and the extent of old-age insurance coverage for employees. In advanced regions, coverage rates are inherently higher, and their susceptibility to fluctuations in enterprise profits is diminished. Conversely, in less developed regions, the impact of enterprise profits on coverage rates is more pronounced. Fifthly, the existence of labor unions exerts a counteractive impact on the influence of enterprise profits over the employee old-age insurance coverage rate. Coverage rates are less contingent on enterprise profits within establishments with active labor unions compared to those without.

The findings of this study hold significant implications for governmental authorities. Firstly, it is recommended to take measures such as reducing the social insurance premium to improve the business environment for private enterprises. Initiatives aimed at enhancing the developmental ecosystem for private enterprises can facilitate a virtuous cycle of enterprise progression. Secondly, the imperative lies in reinforcing enterprise oversight across all regions to ensure the comprehensive discharge of their social insurance commitments, particularly in economically disadvantaged areas. Presently, the policies and institutional frameworks at the regional level evince a lack of uniformity and cohesive implementation. In alignment with principles of legal governance, it becomes indispensable to intricately delineate and harmonize designated participants, contribution benchmarks, and eligibility criteria for old-age insurance benefits for enterprise employees. Thirdly, improve the organizational mechanism of labor unions and workers’ congresses. In a market-oriented economy, investors typically wield significant influence, whereas individual employees hold comparatively diminished sway. Consequently, as societal awareness evolves and employees become increasingly cognizant of their entitlements, labor unions and workers’ congresses must assume an increasingly substantial role in safeguarding employee rights and interests, as well as in labor relations management. Fourth, targeted supervisory measures should be enacted to ensure private enterprises’ adherence to insurance participation requisites. To reduce enforcement expenditures and effectively safeguard the rights of employees, the government should specially regulate those private enterprises in underdeveloped areas, lacking trade unions, and with relatively low profits, as they are more likely to shirk their old-age insurance obligations.

## Data availability statement

The original contributions presented in the study are included in the article/supplementary material, further inquiries can be directed to the corresponding authors.

## Author contributions

WX: Conceptualization, Methodology, Project administration, Writing – review & editing. HM: Formal analysis, Visualization, Writing – original draft. ZP: Data curation, Investigation, Writing – original draft. CP: Supervision, Validation, Writing – review & editing. XQ: Conceptualization, Writing – review & editing.

## Footnotes

^1^ https://cpes.zkey.cc/index.jsp
